# Leveraging glycosylation for early detection and therapeutic target discovery in pancreatic cancer

**DOI:** 10.1038/s41419-025-07517-z

**Published:** 2025-03-31

**Authors:** Tomasz Pienkowski, Katarzyna Wawrzak-Pienkowska, Anna Tankiewicz-Kwedlo, Michal Ciborowski, Krzysztof Kurek, Dariusz Pawlak

**Affiliations:** 1 Clinical Research Center, Medical University of Bialystok, Sklodowskiej MC 24A, Bialystok, Poland; 2 Department of Pharmacodynamics, Medical University of Bialystok, Bialystok, Poland; 3 Department of Gastroenterology and Internal Medicine, Medical University of Bialystok, Bialystok, Poland; 4 Department of Gastroenterology, Hepatology and Internal Diseases, Voivodeship Hospital in Bialystok, Bialystok, Poland

**Keywords:** Biochemistry, Biomarkers, Pancreatic cancer

## Abstract

Pancreatic cancer (PC) remains one of the most lethal malignancies, primarily due to late-stage diagnosis, limited biomarker specificity, and aggressive metastatic potential. Recent glycoproteomic studies have illuminated the crucial role of glycosylation in PC progression, revealing altered glycosylation patterns that impact cell adhesion, immune evasion, and tumor invasiveness. Biomarkers such as CA19-9 remain the clinical standard, yet limitations in sensitivity and specificity, especially in early disease stages, necessitate the exploration of alternative markers. Emerging glycoproteins—such as mesothelin, thrombospondin-2, and glycan modifications like sialyl-Lewis x—offer diagnostic promise when combined with CA19-9 or used in profiling panels. Furthermore, therapeutic strategies targeting glycosylation processes, including sialylation, and fucosylation, have shown potential in curbing PC metastasis and enhancing immune response. Translational platforms, such as patient-derived xenografts and advanced in vitro models, are pivotal in validating these findings and assessing glycosylation potential therapeutic impact. Continued exploration of glycosylation-driven mechanisms and biomarker discovery in PC can significantly advance early detection and treatment efficacy, offering new hope in the management of this challenging disease.

## Facts

- Glycosylation-related biomarkers: biomarkers such as CA19-9, MSLN, and THBS2 offer diagnostic potential, with studies aiming to improve early detection accuracy through combinations with N-glycan profiles.
- Glycosylation-driven metastasis and immune evasion: alterations in sialylation and fucosylation enhance PC cell adhesion, migration, and immune escape, highlighting therapeutic targets for limiting metastasis.
- Therapeutic targeting of glycosylation pathways: inhibitors targeting specific glycosylation pathways (e.g., N-glycosylation in ferroptosis resistance) and the use of translational platforms hold promise for novel therapeutic strategies in PC treatment.

## Open questions

- How can glycoproteomic profiling be standardized across diverse patient cohorts to develop universally applicable biomarkers for early pancreatic cancer detection?
- What are the most effective strategies for therapeutically targeting aberrant glycosylation patterns in pancreatic cancer without disrupting normal glycosylation-dependent cellular functions?
- How does the dynamic regulation of glycosylation patterns contribute to pancreatic cancer progression, and can it be leveraged to monitor treatment response in real-time?

## Introduction

Pancreatic cancer is one of the leading causes of cancer-related fatalities, characterized by a poor prognosis. Approximately 90% of pancreatic tumors are classified as pancreatic ductal adenocarcinoma (PDAC), which has one of the lowest survival rates among all cancers. The median survival for patients diagnosed with pancreatic cancer is around six months, and the five-year survival rate is ~12% [1]. One of the most significant challenges in managing pancreatic cancer lies in its late presentation and the absence of accurate, reliable biomarkers for early detection [Table 1]. Patients frequently exhibit few, if any, noticeable symptoms, which often results in metastases being present at the time of diagnosis. It is estimated that 50–60% of patients have metastatic disease at diagnosis, leaving them without the possibility of surgical intervention with curative intent [2, 3]. Currently, the only curative option available is surgical resection; however, this is feasible in fewer than 20% of PDAC cases. Surgical resection remains the sole curative treatment, yet it is applicable in <20% of cases. Treatment approaches depend on radiological imaging, which facilitates the classification of the disease into three distinct subgroups: local disease, defined by the absence of vascular involvement; regional disease, characterized by varying degrees of vascular involvement and further categorized as either borderline resectable or locally advanced; and metastatic disease [4].

**Table 1:** Clinical relevance in pancreatic cancer.

Biomarker	Sensitivity	Specificity	Clinical relevance	Reference
CA19-9	47–93%	76–90%	Widely used tumor marker; elevated levels indicate pancreatic cancer but can also be elevated in other conditions (e.g., cholangitis, pancreatitis).	[100–102]
CEA (Carcinoembryonic Antigen)	30–88%	81–90%	Less specific for pancreatic cancer; often used in conjunction with CA19-9 for diagnosis and monitoring treatment.	[23, 102, 103]
MUC1	77–96%	56–94%	MUC1 levels can be elevated in various cancers; may help differentiate pancreatic cancer from other malignancies.	[104–106]
CA 50	88%	87–92%	CA 50 is a biomarker that aids in the detection of pancreatic cancer and can be used to monitor disease progression.	[103, 107]
CA 242	68–75%	83–86%	CA 242 has a role in pancreatic cancer diagnosis, showing potential for early detection and monitoring due to its higher specificity than some other markers.	[102, 108]
CA 72-4	58–63%	86–98%	CA 72-4 may assist in distinguishing pancreatic cancer from benign pancreatic conditions, adding value when used alongside other biomarkers.	[109]
CA 125	42–79%	64–96%	CA 125 can serve as a supplementary biomarker in pancreatic cancer, especially useful for monitoring treatment response and detecting recurrence.	[110, 111]
Combination of CA19-9 and CA 72-4	71%	93%	The combination of CA19-9 and CA 72-4 improves diagnostic accuracy and sensitivity for pancreatic cancer compared to either marker alone.	[112, 113]
Combination of CA19-9 and CA 125	88%	78%	Using both CA19-9 and CA 125 together can enhance sensitivity in detecting pancreatic cancer, particularly in identifying advanced disease.	[114]
Combination of CA19-9, CEA, CA 125, and CA 242	91%	94%	A panel of CA19-9, CEA, CA 125, and CA 242 biomarkers significantly improves diagnostic accuracy and helps in differentiating pancreatic cancer from non-cancerous conditions, making it useful for comprehensive screening and monitoring.	[115]

Carcinogenesis in pancreatic cancer is driven by the progressive accumulation of mutations, particularly in key oncogenes and tumor suppressor genes. Notably, mutations in the Kirsten Rat Sarcoma (KRAS) oncogene and loss-of-function alterations in tumor suppressor genes such as TP53, CDKN2A, SMAD3, and SMAD4 are critical in the development of this disease. Additionally, germline mutations in genes like BRCA1, BRCA2, PALB2, ATM, and mismatch repair genes (MLH1, MSH2, MSH6) are associated with an increased predisposition to pancreatic cancer [5, 6]. These genetic changes are paralleled by significant histological alterations within pancreatic ductal cells, leading to the formation of preneoplastic lesions known as pancreatic intraepithelial neoplasia (PanIN). The progression from these early lesions to invasive adenocarcinoma is marked by processes such as acinar-to-ductal metaplasia (ADM), where acinar cells lose their differentiated state and adopt characteristics of ductal cells. Although the exact relationship between ADM and PDAC remains somewhat ambiguous, it has been observed in mouse models where a majority of PanIN lesions originate from acinar cells [7, 8]. Furthermore, ADM is considered a hallmark of pancreatitis, which is recognized as the most significant risk factor for the development of PDAC in humans. Alternative pathways leading to pancreatic carcinoma include intraductal papillary mucinous neoplasm (IPMN) and mucinous cystic neoplasm, with IPMN often exhibiting high frequencies of mutations in the GNAS gene. Despite the identification of these genetic alterations, the therapeutic landscape for pancreatic cancer remains challenging. The most frequently mutated genes—CDKN2A, TP53, KRAS, and SMAD4—currently lack viable pharmacological targets, significantly hindering the development of effective treatment options [6].

The complexity of pancreatic cancer is significantly compounded by its intricate genomic, epigenetic, and metabolic features, leading to numerous activated signaling pathways and intercellular interactions that complicate treatment strategies. The tumor microenvironment presents an additional challenge, characterized by a complex interplay between neoplastic cells and the surrounding stromal components. This interaction not only affects the efficacy of medical treatments but also contributes to the difficulty in achieving successful outcomes [4]. While surgical resection remains the primary curative option, neoadjuvant therapies offer a potential pathway to enhance tumor respectability, thus improving patient outcomes. Newer combinations of drugs and multimodal treatment regimens—implemented in both neoadjuvant and adjuvant settings—have shown promise in extending survival. However, it is important to note that approximately 80% of patients will experience a relapse, ultimately leading to disease progression and mortality [6].

### Glycoproteomics overview

Glycosylation, one of the most common post-translational modifications (PTMs), is a complex enzymatic process, primarily occurring in the endoplasmic reticulum (ER) and Golgi apparatus, which involves the attachment of carbohydrate moieties, glycans, to proteins and lipids. These modifications influence protein structure, stability, and function, thereby impacting various biological processes, including cell signaling, immune response, and cellular regulation [9, 10].

There are two main types of glycosylation in humans - N-glycosylation and O-glycosylation. N-glycosylation involves the attachment of N-acetylglucosamine (GlcNAc) to the nitrogen atom of asparagine (Asn) residues through a β-1N linkage, forming glycoconjugates characterized by a core structure of GlcNAc2-mannose (Man)3. It begins in the endoplasmic reticulum with the formation of a lipid precursor attached to dolichol phosphate, followed by transfer of the oligosaccharide to proteins by oligosaccharyltransferase at Asn-X-Ser/Thr sites. As the protein moves through the Golgi apparatus, a series of specific mannosidases trim and modify the glycan structures, yielding high-mannose, hybrid, or complex N-glycans that play critical roles in protein folding, stability, and cell signaling. In contrast, O-glycosylation occurs primarily on serine (Ser) or threonine (Thr) residues, where sugars such as N-acetylgalactosamine (GalNAc) are added one at a time by polypeptide GalNAc transferases, predominantly in the Golgi. This type of glycosylation contributes to the structural diversity of glycoproteins, particularly mucins, which protect epithelial surfaces from external stress and microbial infections. Both glycosylation types are regulated by the expression of various glycosyltransferases and can be influenced by cellular conditions. In the context of this review, it is worthwhile to read Reily et al. [11] review which offers the broader presentation of other glycosylation types in overall health and disease.

**Fig. 1:**
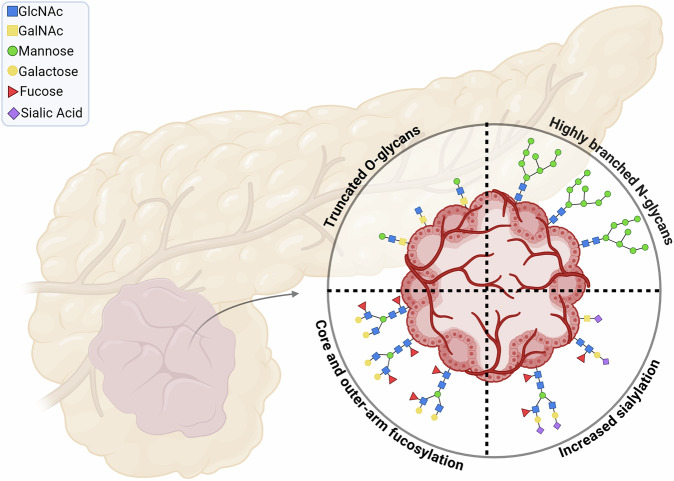
Characteristic glycosylation changes observed in pancreatic ductal adenocarcinoma (PDAC), includes an increase in truncated O-glycans, highly branched N-glycans, and the overexpression of fucosylated and sialylated structures. *Created in BioRender*.

In the context of PDAC, aberrant glycosylation patterns have been closely associated with the malignant transformation and progression of tumor cells [Fig. 1]. Studies have identified specific alterations in glycosylation that are characteristic of PDAC, including an increase in truncated O-glycans, changes in N-glycan branching, and the overexpression of fucosylated and sialylated structures [12]. These glycosylation changes enhance tumor cell survival, proliferation, and invasive abilities, making them key players in cancer progression. For instance, the presence of sialylated glycans, such as those in the sialyl-Lewis family, is linked to increased metastatic potential, highlighting how glycosylation can facilitate tumor spread [13].

**Fig. 2:**
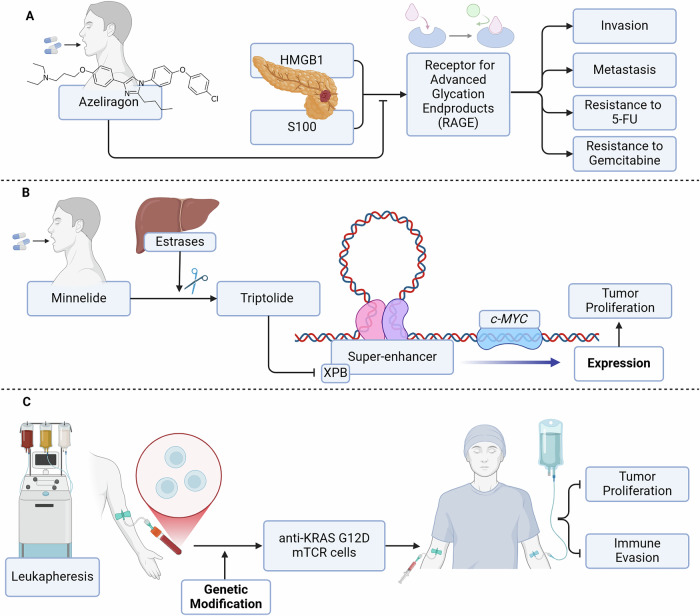
Methodology of selected active clinical trials. **A** Azeliragon binds competitively with RAGE ligands, such as S100 and HMGB1, inhibiting their interaction with the receptor. This action halts invasion, metastasis, and resistance to chemotherapeutic agents like 5-FU and gemcitabine, improving treatment efficacy in cancer therapy. **B** the prodrug Minnelide is cleaved by liver esterases into its active form, triptolide, which then inhibits XPB, a component of the transcription machinery involved in superenhancer activity. This inhibition reduces MYC expression, thereby impacting tumor proliferation and progression. **C** T cells are collected through leukapheresis and genetically modified to specifically target and kill pancreatic cancer cells expressing the KRAS G12D mutation. When combined with chemotherapy, this approach may enhance the immune response while potentially reducing tumor burden and overcoming the resistance mechanisms typically seen in pancreatic cancer. *Created in BioRender*.

Moreover, glycosylation impacts immune recognition and evasion, which are critical in the tumor microenvironment. Tumor cells often exploit altered glycosylation to escape immune surveillance, promoting an immunosuppressive environment that further enhances tumor progression. The interaction between tumor cells and surrounding stromal components, including cancer-associated fibroblasts, is also influenced by glycosylation [14]. These interactions create a complex tumor microenvironment that fosters angiogenesis, immune evasion, and resistance to therapy, complicating treatment strategies for PDAC. The importance of glycosylation in PDAC is highlighted by the fact that many established biomarkers are glycan-based. For instance, CA19-9, a well-known clinical blood biomarker for pancreatic cancer, detects the epitope of sialyl-Lewis on mucins and other adhesive molecules [13]. MUC1 is a heavily O-glycosylated protein characterized by extensive carbohydrate side chains attached to its serine and threonine residues, which are further modified with terminal sialic acid residues. This dense glycosylation, including the sialylation, contributes to MUC1 protective and structural functions, especially on epithelial surfaces. The terminal sialic acids play a key role in modulating immune recognition and cellular interactions, often helping cancer cells evade immune detection and promoting tumor progression when MUC1 is overexpressed or abnormally glycosylated in cancerous tissues [15] This highlights the diagnostic potential of glycosylation changes in PDAC, as many patients present with altered glycan profiles even before the onset of overt disease. The ability to detect these changes early may aid in the diagnosis and monitoring of PDAC, offering a critical advantage as pancreatic cancers are known for their late presentation. Some glycation end products are investigated in clinical trials on pancreatic cancer [Fig. 2] [Table 2].

**Table 2:** Clinical Trials of Glycosylation End Products on Pancreatic Cancers.

Clinical Trial No.	Phase	Title	Case	Description	Status
NCT04896073	Pahse II	Superenhancer Inhibitor Minnelide in Advanced Refractory Adenosquamous Carcinoma of the Pancreas	Pancreatic Ductal Adenocarcinoma variant: Advanced Refractory Adenosquamous Carcinoma of the Pancreas	Treatment with Minnelide, prodrug of triptolide, an anticancer agent that targets cancer resistance through several mechanisms.	Recruiting
NCT05766748	Pahse I/II	Study of Effect of Azeliragon in Patients Refractory to Prior Treatment of Metastatic Pancreatic Cancer	Metastatic Pancreatic Cancer in Patients Refractory to Prior Treatment	Treatment with Azeliragon, an orally administered inhibitor of Receptor for Advanced Glycation End-products (RAGE)	Recruiting
NCT03451773	Phase IB/II	M7824 (MSB0011359C) in Combination With Gemcitabine in Adults With Previously Treated Advanced Adenocarcinoma of the Pancreas	Advanced Pancreatic Ductal Adenocarcinoma	M7824 (MSB0011359C) in Combination With Gemcitabine. Blockade of a pathway that prevents the immune system from effectively fighting cancer	Terminated - treatment-related death
NCT03300843	Phase II	Ability of a Dendritic Cell Vaccine to Immunize Melanoma or Epithelial Cancer Patients Against Defined Mutated Neoantigens Expressed by the Autologous Cancer	Pancreatic Cancer, Gastrointestinal Cancer, Breast Cancer, Ovarian Cancer, Melanoma	Dendritic cell vaccine	Terminated - slow accrual
NCT03745326	Phase I/II	Administering Peripheral Blood Lymphocytes Transduced With a Murine T-Cell Receptor Recognizing the G12D Variant of Mutated RAS in HLA-A*11:01 Patients	Pancreatic Cancer, Gastrointestinal Cancer, Gastric Cancer, Colon Cancer, Rectal Cancer	Determination oif anti-KRAS G12D mTCR PBL is capable of the regressing of tumors harboring the RAS G12D mutation	Recruiting
NCT03785210	Phase II	Nivolumab (Anti-PD1), Tadalafil and Oral Vancomycin in People With Refractory Primary Hepatocellular Carcinoma or Liver Dominant Metastatic Cancer From Colorectal or Pancreatic Cancers	Patients With Refractory Primary Hepatocellular Carcinoma or Liver Dominant Metastatic Cancer From Colorectal or Pancreatic Cancers	Treatment with Nivolumab (Anti-PD1), Tadalafil and Oral Vancomycin	Completed - no data published
NCT04034238	Phase I	Mesothelin-Targeted Immunotoxin LMB-100 in Combination With Tofacitinib in Persons With Previously Treated Pancreatic Adenocarcinoma, Cholangiocarcinoma and Other Mesothelin Expressing Solid Tumors	Patients With Previously Treated Pancreatic Adenocarcinoma, Cholangiocarcinoma and Other Mesothelin Expressing Solid Tumors	Mesothelin-Targeted Immunotoxin LMB-100 in Combination With Tofacitinib	Completed

Despite glycosylation potential as a biomarker and therapeutic target, current clinical applications are limited. Traditional proteomics often fails to identify glycoprotein markers due to challenges in quantifying glycopeptides and the complexity of glycosylation. However, advances in glycoproteomic methods, such as lectin affinity chromatography and mass spectrometry, offer promise for uncovering reliable biomarkers. These techniques enable detailed analysis of glycan structures and their role in cancer progression, potentially identifying novel therapeutic targets [10, 16].

Rationale for focus is a deeper understanding of the glycosylation landscape in pancreatic cancers could point us to the new diagnostic biomarkers and therapeutic targets, ultimately improving patient outcomes in this, highlighting again, late presenting malignancy.

## Glycosylation changes and early detection in pancreatic cancer

**Fig. 3:**
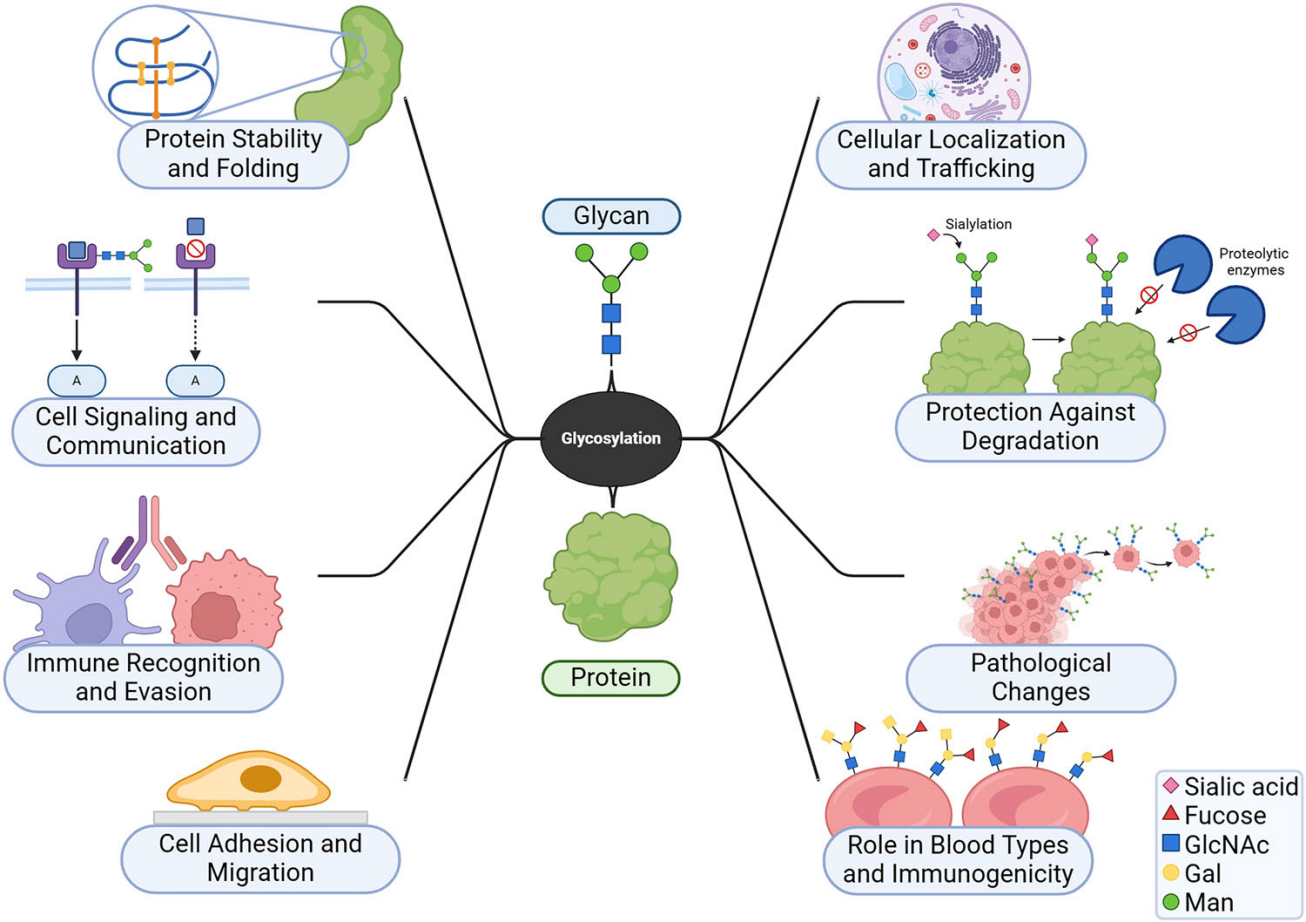
Glycosylation in cellular function and health. As a critical post-translational modification, glycosylation influences protein stability and folding, ensuring proper conformation and preventing misfolding. It governs cellular localization and trafficking, directing proteins to appropriate compartments while protecting them against degradation by shielding them from proteolytic enzymes. Glycosylation is pivotal in cell signaling and communication, modulating responses and interactions. It also impacts immune recognition and evasion, aiding in immune modulation and pathogen survival. Additionally, glycosylation facilitates cell adhesion and migration, essential for tissue organization and repair, while alterations in glycosylation patterns are linked to pathological changes, such as cancer progression and chronic inflammation. Finally, it determines blood group antigens and immunogenicity, influencing transfusion compatibility and immune responses. This underscores glycosylation’s significance in maintaining cellular homeostasis and its potential as a therapeutic target in disease contexts. *Created in BioRender*.

Glycosylation influences cancer progression by modifying several key cellular processes. The overexpression of branched N-glycans disrupts epithelial cadherin-mediated cell-cell adhesion, promoting tumor cell dissociation and invasion [Fig. 3]. Additionally, modifications of integrins with branched N-glycans, truncated O-glycans, or sialylated structures alter tumor cell interactions with the extracellular matrix, facilitating cell migration. Sialyalted glycoproteins were already associated with pancreatic cancer cell adhesion and metastasis [17]. The altered expression of proteoglycans and their glycosaminoglycan chains affects the activation of tyrosine kinase receptors, while changes in glycosylation of growth factor receptors and gangliosides modulate signal transduction pathways, enhancing tumor growth and proliferation. Moreover, glycans and their corresponding lectins regulate inflammation and immune responses, further supporting tumor survival and immune evasion [18].

Classical diagnostic biomarkers for pancreatic cancer include several key markers, each with specific limitations and benefits. CA19-9 is the most commonly used biomarker, and a glycoprotein, despite its low specificity and sensitivity, especially in early-stage disease and in patients who lack the Lewis antigen (~5–10% of the population), which is required for CA19-9 expression. Currently, the only biomarker accepted by the FDA and the National Comprehensive Cancer Network guidelines [19, 20]. CEA (Carcinoembryonic Antigen) is another marker that, when used alone, has limited specificity but is often combined with CA19-9 to improve diagnostic accuracy [21]. However, CEA levels can also be elevated in other gastrointestinal cancers and inflammatory conditions, making it a less reliable standalone marker [22, 23]. SPan-1, a serum biomarker that has shown promise in detecting PDAC, sometimes demonstrates higher sensitivity than CA19-9, though it is not as widely utilized in clinical practice due to limited clinical validation, concerns about sensitivity and specificity, lack of standardization, and preference for more established biomarkers like CA19-9 [24]. Recently, thrombospondin-2 (THBS2) has been identified as a promising diagnostic biomarker when combined with CA19-9 to improve the early detection of PDAC [25].

Besides glycoproteins mentioned above, more glycoprotein biomarkers for pancreatic cancer have been explored in pursuit of improved diagnostic accuracy, though many face challenges in clinical application. The POA marker, developed in the 1960s, showed initial promise for detecting oncofetal antigens in serum but suffers from standardization issues and variability in sensitivity (68–81%), limiting its usefulness in early diagnosis, particularly as it is often pathological in other hepato-biliary diseases [26, 27]. Cytokeratins, such as TPA and TPS, provide insights into cell proliferation but have demonstrated sensitivities (48–96% for TPA, and 50–98% for TPS) and specificities (22–70% for TPS) that are generally inferior to CA19-9 [28, 29]. Mucins, including Du-PAN 2, SPan-1, and CAM17.1, represent another class of glycoprotein biomarkers; Du-PAN 2 is notable for recognizing a tumor marker in Lewis a-b-individuals with a sensitivity of 48–72%, while SPan-1 initially showed promise with a sensitivity of 82–92%, although it does not significantly enhance the accuracy of CA19-9. CAM17.1, which has a reported sensitivity of 78% and specificity of 76% [26]. Emerging glycoprotein biomarkers show promise for the early detection of pancreatic cancer (PC), with several notable candidates highlighting their potential clinical utility. Leucine-rich alpha-2 glycoprotein 1 is associated with poor survival and advanced tumor stages, promoting the viability and invasion of pancreatic tumor cells [30, 31]. Matrix metalloproteinases (MMPs), particularly MMP-9, which is a glycoprotein involved in the degradation of extracellular matrix components, play a crucial role in cancer invasion and metastasis but are considered inferior markers compared to CA19-9. Tissue inhibitors of metalloproteinases (TIMPs), such as TIMP-1, have shown potential with a sensitivity of 47.1% and specificity of 69.2% [32, 33]. Transthyretin, a glycoprotein carrier of thyroid hormones, is elevated in PDAC patients, indicating a sensitivity of 90.5% and specificity of 47.6% [34]. Intercellular adhesion molecule 1, also a glycoprotein, has demonstrated good diagnostic performance with an AUC of 0.851 at a specific cutoff [35, 36]. Lastly, osteoprotegerin, another glycoprotein, is upregulated in cancerous pancreatic tissue and is particularly elevated in patients with new-onset diabetes, suggesting its relevance in early detection [37, 38]. Despite their potential, these glycoprotein biomarkers require further validation and standardization for effective clinical use in diagnosing pancreatic cancer.

In recent research strictly focusing on glycosylation or glycosylated proteins in pancreatic cancer, which may pose therapeutic target capabilities there are a few that may be suggested as worth further investigation. Guerrero et al. [13] investigated PDAC in search of a diagnostic biomarker using the glycoproteomic approach presented novel potential protein carriers for sialyl-Lewis x in their glycans. SLex belongs to the Lewis antigens that are identified as tumor-associated antigens, which are distinctly expressed in a large number of tumor tissues, but not in their corresponding non-tumor ones. The increase in sLex in the tumor tissue.

Glycoproteins can be reflected in the tumor proteins secreted to the serum, and it was described, earlier by the same group, several biomarkers expressing sLex in serum proteins of advanced PDA patients such as ceruloplasmin. However, these reported glycoproteins carrying sLex were acute-phase proteins, mainly liver-derived [39]. But in the follow-up study, they proposed a pancreatic tumor-derived glycoproteomic sLex carrier, MFAP4, an extracellular glycoprotein [40]. Moreover, Guerrero et al. [13] revealed MFAP4 absence in the cell lysates and conditioned media of seven analyzed PDA cell lines but its presence in the pancreatic extracellular matrix of PDA patient tissues. Also, MFAP4 protein levels are higher in tissue samples of PDA compared to non-tumor pancreas [13], but its role in malignancy across other carcinomas remains unclear due to inconsistent literature; while some studies report MFAP4 upregulation in aggressive prostate cancer [41], others show downregulation compared to benign prostate hyperplasia [42].

Currently, there are no reliable biomarkers for early diagnosis of pancreatic cancer, but Duran et al. [12] proposes a mesothelin (MSLN) as a potential candidate. MSLN is a glycoprotein neo-expressed and released from the cell membrane in pancreatic cancer to reach the bloodstream. It mediates cellular adhesion through binding to another glycoprotein, MUC16, in cancer, and contributes to key processes such as resistance to cell death, proliferation, invasiveness, metastasis, angiogenesis, and epithelial-to-mesenchymal transition [43]. MSLN has a highly restricted expression pattern, being predominantly found in mesothelioma or ovarian cancer. In healthy tissues, its expression is limited to the pericardium, peritoneum, and pleura [44, 45]. Duran et al. [12] revealed that MSLN N-glycans are predominantly complex-type, highly branched, and feature core fucosylation and β2,3-linked sialic acid (SA). The β2,3-SA, linked to the immunosuppressive properties of pancreatic cancer, was the primary glycoform in most investigated cell lines. Notably, core fucosylated MSLN (Cf-MSLN) was found to be reduced in tumor tissues and serum compared to healthy controls, suggesting its potential as a more specific biomarker for pancreatic cancer than total MSLN levels. These findings point to the importance of cancer-specific glycoforms for further diagnostic purposes in pancreatic cancer. The abovementioned MSLN was also identified as an elevated glyco-signature in a recent study by Lih et al. [46], along with increased levels of POSTN, THBS1, and immune-related proteins like CEACAM1 and VNN1, all of which are suggested as potential diagnostic biomarkers for PDAC. In addition, plasma samples of PDAC patients also present elevated levels of glycosylated galectin-3 binding proteins (LGALS3BP) [47]. The abundant levels of LGALS3BP in PDAC were previously reported by Pan et al. [48], alongside mucin-5AC (MUC5AC), carcinoembryonic antigen-related cell adhesion molecule 5 (CEACAM5), and insulin-like growth factor binding protein (IGFBP3). However, this study was conducted on tissue samples. Portfield et al. [49] took an alternative approach to serum analysis by investigating pancreatic ductal fluid, which, unlike serum, requires an endoscopic procedure for collection. Their study found several proteins with elevated levels, including AMYP, PRSS1, GP2-1, CCDC132, REG1A, REG1B, and REG3A, alongside one protein with decreased expression, LIPPR2—all associated directly or indirectly with the secretory pathway. A comprehensive analysis of the N-linked glycome in pancreatic ductal fluid further revealed unexpected clustering of patient samples into distinct subgroups enriched in sialylation, fucosylation, or a mix of both. This clustering highlights the heterogeneity of glycosylation patterns across individuals, underscoring the need for caution when interpreting glycomic and glycoproteomic profiles, which shows the importance of maintaining detailed patient records and proceeding with screening methods which would further allow us to cluster these patients on known common signatures for further evaluation, which glycosylation pattern they follow. The abovementioned possesses huge potential for diagnostic biomarkers. However, this is a 2014 study with no follow-up.

Aronsson et al. [50] aimed to develop a specific biomarker panel by combining CA19-9 with N-glycan profiles of selected serum glycoproteins for the early detection of pancreatic cancer. Their study demonstrated that stage 1 pancreatic cancers could be detected with high accuracy using a biomarker panel that included B7-1, IL-17E, DR6, and CA19-9, achieving an AUC of 0.988, with 100% sensitivity at 90% specificity. This combination significantly improved diagnostic accuracy compared to using CA19-9 alone. Furthermore, the same panel effectively discriminated all stages of pancreatic cancer from healthy controls, with an AUC of 0.974 and a sensitivity of 96% and a specificity of 90%. Notably, the overexpression of B7-1 and B7-H proteins emerged as both predictive and detection markers, offering promise for enhancing early diagnosis [51, 52].

Another screening panel for early pancreatic cancer detection was investigated by Mann et al. [53]. Identified was a total of 80 N-linked glycans by analyzing pancreatic cyst fluid. Of particular interest in this study were complex N-glycans containing two to six fucose residues. Following lectin enrichment, triacylglycerol lipase and pancreatic alpha-amylase were notably elevated, showing a 20- and 22-fold increase in abundance, respectively. Both enzymes have previously been used as potential biomarkers for pancreatic disease. The literature supports increased fucosylation in serum proteins as potential biomarkers for cancers. For instance, core fucosylation of ribonuclease 1 and both core and outer arm of haptoglobin have been reported as potential indicators [54–56]. Furthermore, di- and trifucosylated lactosamine extensions on glycans have been observed in tissues and serum not only of pancreatic cancer patients but also in other cancer types, suggesting these glycan modifications may be indicative of malignancy more broadly [57, 58].

Despite promising findings, further studies are needed to assess the diagnostic feasibility of this glycosylation pattern in larger cohorts. Practical challenges, such as the high cost of lectin enrichment and LC-MS/MS analysis, may limit accessibility, especially in hospitals without specialized equipment. This could require external sample processing, delaying diagnostics, and increasing costs. However, investigating fucosylation patterns could lead to novel therapies, as triacylglycerol lipase and alpha-amylase play key roles in cell metabolism and proliferation [59, 60]. Characterizing their glycosylation profiles may offer therapeutic targets to reduce their activity in cancer cells, though this remains theoretical and requires further research.

On the other hand, glycoproteomics research has also focused on identifying prognostic factors for pancreatic cancer. For instance, Lu et al. [61] conducted a study demonstrating that fucosylated SERPINA1 could distinguish between non-metastatic pancreatic cancer patients and a control group (patients with gallstones). Importantly, its presence was associated with poorer overall survival, suggesting its potential as a prognostic marker. However, whether fucosylated SERPINA1 can also serve as a reliable biomarker for early detection remains to be validated in future studies.

## Therapeutic target discovery and translational implications

Lu et al. [62] translated their data from cell models to clinical tissue samples by developing a quantitative N-glycoproteomics approach that is site- and structure-specific, which allowed them to identify potential N-glycoprotein cancer biomarkers at the intact N-glycopeptide level. Remarkably, the study revealed differential expression patterns that were both site- and structure-specific. They observed variations not only in site isomers—where the same N-glycan is present at different sites on the same protein—but also in structure isomers, such as variations in sialic acid linkages and fucose positioning. These PDAC tissue samples, compared to control samples, presented 52 differentially expressed intact N-glycopeptides, comprising 38 that were upregulated and 14 that were down-regulated.

N-glycan profiling in cancer cells has revealed notable alterations in the glycosylation patterns of secreted glycoproteins also in other studies. Xu et al. [63] derived these profiles from the primary tumor of MIA PaCa-2 pancreatic cancer cells. In parallel, changes in glycan structures were observed at specific glycosylation sites in human sera, which contain numerous secreted glycoproteins. These findings suggest that tumor-specific glycosylation patterns could serve as potential biomarkers for pancreatic cancer, offering a non-invasive approach to disease detection and monitoring. Additionally, treatment with AMG-510, a small molecule inhibitor targeting the KRAS G12C mutation, was found to significantly reduce the glycosylation levels in MIA PaCa-2 cells. This indicates that KRAS is likely involved in regulating cellular glycosylation processes. The reduction in glycosylation associated with AMG-510 treatment suggests that inhibiting KRAS not only disrupts tumor growth but also interferes with glycosylation pathways, which may further contribute to its anti-tumor effects.

While, fucosylation profile was investigated by Min et al. [64], who initially examined it in a cell line model before translating their findings to serum samples. Their study revealed a significant dysregulation of core fucosylation glycopeptides in preoperative versus postoperative serum samples from patients with PDAC. Notably, the core fucosylation modifications of specific glycopeptides, including BCHE_N369, CDH5_N112, and SERPIND1_N49, were identified as potential prognostic markers. This study highlights the significance of fucosylation in pancreatic cancer screening, further reinforcing the importance of fucosylation discussed in the previous section of this manuscript.

Ma et al. [65] investigated the mechanism of N-glycosylation in PDAC ferroptosis resistance. Aberrant N-glycosylation of the protein 4F2hc, catalyzed by the glycosyltransferase B3GNT3, stabilizes its membrane localization and facilitates xCT trafficking, forming the system X_C_–, which promotes cystine uptake and glutathione peroxidase 4 activation, thus enabling PDAC cells to resist ferroptosis. In contrast, inhibiting N-glycosylation (using enzymes like PNGase F, tunicamycin, or knockout of B3GNT3) destabilizes 4F2hc and disrupts the system X_C_–, making PDAC cells more sensitive to ferroptosis. The study reveals that high expression of 4F2hc correlates with ferroptosis resistance and identifies Asn365 as a significant glycosylation site in this process. Additionally, B3GNT3 was found to play a critical role in ferroptosis resistance and PDAC progression, with its knockout suppressing tumor proliferation and migration, which suggests that targeting the N-glycosylation pathway, particularly through the inhibitor tunicamycin, could sensitize PDAC cells to ferroptosis and improve treatment outcomes, though further research is needed to refine such approaches due to the potential cytotoxicity of off-target effects in translational platforms.

Miayara et al. [66], on the other hand, explored potential secreted biomarkers in pancreatic cancer through glycoproteomic analysis of three pancreatic cancer cell lines (BxPC-3, MIA PaCa-2, and PANC-1). They identified prosaposin (PSAP), a protein with various neurotrophic functions, as highly expressed across the cell lines. However, knockdown experiments revealed that PSAP depletion did not alter tumor proliferation or migration, ruling it out as a direct therapeutic target. Despite this, high PSAP expression was linked to poor prognosis, as tumors with elevated PSAP levels exhibited significantly reduced CD8+ T-cell infiltration compared to tumors with lower PSAP expression. PSAP stimulation was also found to decrease the proportion of CD8+ T cells in peripheral blood mononuclear cells. In an orthotopic transplantation model, PSAP knockdown via shRNA led to a notable increase in CD8 + T-cell infiltration, which was followed by a significant reduction in tumor volume compared to the control group, thereby promoting the progression of PDAC. Furthermore, transitioning from in vitro studies to an orthotopic transplantation model reveals additional insights, emphasizing the importance of studying tumor-immune interactions across different experimental systems to uncover deeper mechanistic understanding and therapeutic implications.

Li et al. [67], employed integrated quantitative N-glycoproteomics to explore the synergistic anti-tumor effects of aspirin and gemcitabine in pancreatic cancer cell lines PANC-1 and BxPC-3. The results demonstrated that aspirin significantly enhanced the inhibitory effects of gemcitabine on cell viability. Through an integrated multiomics approach, their analysis of intact N-glycopeptide profiles revealed two distinct trends linked to aspirin addition, showing a strong correlation between N-glycosylation changes and the synergistic anti-tumor effects observed. Specifically, the dynamic regulation of sialylation and the presence of high-mannose glycoforms on extracellular matrix-related proteins, such as LAMP1, LAMP2, and ITGA3, were identified as critical factors in enhancing gemcitabine anti-tumor activity and addressing drug resistance in pancreatic cancer cells. Overall, this provides valuable insights into N-glycosylation processes that could inform future research on mechanisms of drug resistance in pancreatic cancer, linking it with changing glycosylation patterns.

While, Masuda et al. [68] focused on creating a next-generation glycan antibody for fucosylated Mac-2-binding protein (Mac-2), a glycoprotein, which increased levels were already correlated with other cancers (eg. liver). Mac-2 isomers are abundantly present in pancreatic cancer patient’s serum [69]. They used fucosylation-deficient HEK293T cells to prepare reference Mac-2 antigens. The 19-8H monoclonal antibody obtained with our screen recognized 70 K Mac-2, which is a C-terminus-truncated product, rather than specifically recognizing fucosylated Mac-2. 19-8H mA showed strong cell surface staining of anticancer drug-resistant cancer cells [68]. Which may show potential in further research. Overall monoclonal antibodies in immunotherapies of pancreatic cancer will be an important step in PDAC therapy, marking a significant advancement in treatment options. However, as of now, no antibody-based therapies have received approval for clinical use in patients with pancreatic cancer [70]. Nevertheless, preclinical and clinical research exploring the efficacy of monoclonal antibodies in this context, as well as the factors contributing to the limited response rates to antibody therapies, can be investigated in another review [71].

Jiang et al. [72] investigated the N-glycosylation gene MGAT1, expressed in macrophages, acinar, and epithelial cells within the TME. MGAT1 expression and glycosylation are lower in tumor tissues than in normal tissues, correlating with tumor progression and poorer prognosis in PDAC. Functional experiments showed that MGAT1 overexpression inhibits PDAC cell proliferation, migration, and invasion, suggesting its role as a tumor suppressor. Spatial transcriptomics and single-cell RNA sequencing revealed that MGAT1 affects cell communication and immune infiltration, making it a potential therapeutic target.

### O-Glycosylation targeting and role of mucins

Glucosaminyl (N-Acetyl) Transferase 3 (GCNT3) is a glycosyltransferase that plays a key role in the biosynthesis of core 2 O-glycans. By adding N-acetylglucosamine to glycoproteins, GCNT3 facilitates the production of mucin-type O-glycans, which are essential for the structural integrity and diverse functional roles of mucins. These high molecular weight glycoproteins are abundantly expressed in PDAC, where they contribute significantly to tumor progression and immune evasion [73].

Aberrant GCNT3 expression is linked to aggressive tumor behaviors in PDAC. Overexpression promotes excessive mucin production, forming a dense barrier that blocks drug penetration and suppresses immune cell infiltration, aiding immune evasion. GCNT3 also interacts with oncogenic pathways like the β-catenin/MUC4 axis, driving PDAC progression by promoting EMT, enhancing stemness, and increasing proliferation. Inhibitors like talniflumate have shown the potential to disrupt GCNT3 activity[74]. Preclinical studies demonstrated that talniflumate significantly reduces mucin production, enhances T-cell activity, and sensitizes tumors to chemotherapeutics like gemcitabine and nab-paclitaxel [74, 75].

Targeting GCNT3 offers benefits beyond disrupting mucin biosynthesis by modulating the TME in PDAC. Inhibiting GCNT3 reduces mucin production, weakening the protective barrier around tumor cells, improving drug delivery, and restoring immune cell activity. This disruption counteracts the immunosuppressive TME in PDAC, enhancing therapeutic efficacy. Additionally, GCNT3 inhibition may downregulate mucins like MUC1 and MUC5AC, which are linked to tumor progression and metastasis. Combining GCNT3 inhibitors with chemotherapies like gemcitabine and nab-paclitaxel may increase tumor sensitivity and address drug resistance. Pairing them with immune checkpoint inhibitors could improve immune surveillance and improve outcomes. Further, combining these inhibitors with agents targeting pro-inflammatory pathways may disrupt tumor-promoting inflammation, weakening cancer cell interactions with the microenvironment [76].

The focus on mucins extends to sialylation’s role in modulating mucin properties and tumor progression. Sialylation and mucin O-glycosylation are interconnected, with sialic acid residues added to mucin glycans, stabilizing them and preventing degradation. This modification enhances mucin functions, including lubrication, epithelial protection, immune modulation, and hydration. However, dysregulated sialylation, such as excessive α2,6 sialic acid, is common in cancers. Rodriguez et al. [77] showed that hypersialylation in PDAC promotes immune evasion by enabling interactions between α2,3-sialylated glycans and Siglec-7/9 receptors on myeloid cells, driving immunosuppressive TAM differentiation. Blocking Siglec-7/9 or removing sialic acids reduces TAM differentiation, while α2,3-sialic acid dendrimers modulate macrophage phenotypes via Siglec-9, enhancing immunosuppressive markers.

ST6GAL1, a key enzyme in sialylation, has been linked to PDAC progression. Bhalerao et al. [78] showed that ST6GAL1 enhances cancer stem cell characteristics and tumor-initiating potential, leading to more aggressive disease. This enzyme also activates developmental pathways, such as Wnt, Notch, and Hedgehog, and promotes ADM, a precursor to neoplastic transformation. Furthermore, ST6GAL1 activates the epidermal growth factor receptor, supporting KRAS-driven tumorigenesis and cancer cell survival.

Increased sialylation enhances adhesion and metastatic potential in PDAC cells. In the SW1990 cell line, heightened sialylation promotes CD44-mediated adhesion to selectins and integrin-driven mobility on extracellular matrix components like collagen and fibronectin [17]. Controlling sialylation could reduce metastasis and invasiveness. Testing sialylation inhibitors in PDX or organoid models could provide real-time data on their effects, offering a potential therapeutic strategy to slow progression and improve outcomes.

## Future directions challenges

Future directions should consider, beside continuous investigation of new pancreatic cancer biomarkers and therapeutic targets, a use of a comprehensive glycobiology database to store and use data for further search of glycoproteomics data in case of diagnostic and monitoring biomarkers, and potential therapeutic targets. There are already few databases, each of its own purpose.

### Bioinformatics tools and databases

One of them is GPSeeker, a specialized software tool designed for the analysis and identification of glycoproteins and their glycan structures through mass spectrometry data [79]. It efficiently identifies glycoproteins from complex biological samples and allows users to annotate glycan structures based on mass spectrometric measurements. The software integrates with various bioinformatics tools and databases, enhancing its utility in glycoproteomic studies. It also offers customization options for parameter settings, comprehensive database support, and visualization tools to help interpret and present findings effectively. GPSeeker is valuable for applications such as biomarker discovery, drug development, and basic research, allowing researchers to explore glycosylation patterns and their functional implications in biological systems.

Complementing GPSeeker, GlyGen is a specialized database focused on glycoproteomics and glycomics, providing extensive resources on glycan structures, glycoprotein annotations, and associated biological data [80]. GlyGen enables researchers to visualize complex glycan structures and analyze glycomic data through a user-friendly interface, making it particularly valuable for studying the roles of glycans in various biological processes and diseases. Similarly, GlycoMod serves as an essential online tool that allows researchers to predict glycan compositions based on mass spectrometry data. By inputting mass spectrometry results, users can identify potential glycan structures and modifications present in their samples. GlycoMod aids in the analysis of glycoproteins and provides a mechanism for validating experimental results, thereby enhancing the reliability of glycoproteomic studies and streamlining the research process.

In addition to these tools, GlycoSuiteDB is dedicated to the structural and functional analysis of glycoproteins, offering detailed information on glycosylation sites, glycan structures, and their biological activities [81]. Researchers can utilize GlycoSuiteDB to explore the glycoproteome in depth, assess glycosylation patterns, and understand the implications of these modifications in various biological contexts, including disease mechanisms and therapeutic targets. Along similar lines, dbGlycO is a comprehensive database that gathers information on glycan structures, modifications, and their associated biological functions. It serves as a valuable resource for researchers interested in glycobiology, providing tools for analyzing glycan-related data and facilitating the exploration of glycan functions in different biological contexts. dbGlycO supports research by linking glycan structures to their roles in cell signaling, immune response, and disease progression [82].

Lastly, GlycoData and GlyTouCan offer a wealth of information on glycan structures and their relationships to various biological processes serving as repository databases [83, 84]. Each provides analytical data for glycan analysis and visualization, allowing researchers to access detailed information about glycan compositions and modifications. These types of databases are particularly useful for understanding the role of glycans in cellular interactions, signal transduction, and disease mechanisms, thereby enhancing our knowledge of glycobiology. Also, GPNotebook is a Python package that compiles intact glycopeptides identified in different cancers by the Clinical Proteomic Tumor Analysis Consortium and provides analytical tools for detailed glycopeptide characterization. GPnotebook offers functions such as statistical profiling, differential expression analysis, glycosylation subtype classification, exploration of glycosylation-phosphorylation interactions, survival analysis, and assessment of glycosylation enzymes. GPnotebook was successfully implemented in a study on PDAC, validating its capabilities and highlighting the potential of IGPs for identifying cancer-specific alterations and subtypes [85]. Additionally, MS-PyCloud is an open-source, cloud-based pipeline with a graphical user interface for analyzing LC-MS/MS data. Key features of MS-PyCloud include data integrity validation, MS/MS database searching for spectral assignment, false discovery rate estimation, protein inference, identification of PTMs, and quantification of specific modified peptides and proteins. By incorporating open-source software tools with thorough testing and versioning, MS-PyCloud ensures transparency and reproducibility in data analysis [86].

Together, these databases and tools serve as invaluable resources for researchers in the field of glycoproteomics, providing data and functionalities for advancing our experimental results on the complex roles of glycosylation in health and disease.

### Future directions

Choosing the right platform for cancer research is essential for gaining useful insights and advancing treatment options. Researchers have several choices, including cancer cell lines, organoids, and PDXs, each with its own strengths and weaknesses [87, 88]. Cancer cell lines are favored for their cost-effectiveness and suitability for large-scale drug testing. However, they often fail to replicate the complex tumor environment and can lose key characteristics over time. In contrast, organoids offer a more accurate model that captures tumor diversity and supports personalized medicine, but they struggle with nutrient delivery and lack immune system interactions [75]. PDXs closely mimic human tumors and preserve genetic and physical traits in a three-dimensional setting, but they are expensive, time-consuming, and may eventually lose their human tissue components.

Given these limitations, it is clear that no single model can fully capture the complexities of cancer biology, prompting researchers to seek new approaches to improve preclinical models. One major challenge in finding effective treatment targets is that existing models often fail to accurately represent tissue diversity, interactions between tumors and surrounding tissues, and the role of the immune system. To overcome these challenges, there is increasing use of advanced methods like glycoproteomics, next-generation sequencing, and multiomics approaches to address them [9, 10, 89–94]. These technologies help reveal the complex molecular details of tumors and provide a better understanding of their biology. Huang et al. [95] introduced TMEPro, a breakthrough approach for high-resolution profiling of glycosylated secreted and plasma membrane proteins in the TME. By combining spatial and temporal proteomic analyses, it offers a comprehensive view of intercellular signaling between cancer and stromal cells. This method identified key signaling pathways, such as the PDGFR–PTPN11–FOS axis, and regulatory mechanisms like the AXL ectodomain, crucial for tumor progression and immune evasion in PDAC. TMEPro reveals glycosylation-specific alterations driving PDAC, providing insights into potential targets for precision therapies. Furthermore, hybrid models like assembloids and the use of humanized mice offer exciting new possibilities to improve model accuracy and relevance to therapy [96–99]. By combining these innovative strategies, the cancer research community can make significant strides toward identifying effective treatment targets that truly reflect the complexities of human tumors, ultimately leading to better outcomes for patients.

However, the use of glycoproteomic data for therapeutic purposes holds great potential, but it is limited by several challenges. One of the main problems is the high cost of advanced glycoproteomic technologies, which restricts accessibility for many research institutions and clinical applications. Techniques such as mass spectrometry and high-throughput glycan analysis require specialized equipment, skilled personnel, and time-intensive processing, all of which contribute to elevated costs. Additionally, the availability of these technologies is limited, especially in standard clinical settings, where the focus is often on established diagnostic tools. Another issue is the complexity of glycoproteomic data, which demands robust computational infrastructure and expertise for data analysis and interpretation. As a result, while glycoproteomic insights have the potential to improve personalized medicine and improve disease diagnosis and treatment, these current limitations halt their widespread clinical adoption.

## Conclusion

PC glycoproteomics and glycosylation studies have provided essential insights into potential diagnostic biomarkers and therapeutic targets. This review highlights the significance of glycosylation patterns, including sialylation and fucosylation, in cancer progression, immune evasion, and metastatic potential. Current studies identify biomarkers, such as CA19-9 and emerging glycoproteins, while innovative techniques like N-glycoproteomics refine biomarker specificity for early PC detection. Exploring glycosylation in PC offers promising insights for identifying therapeutic targets and understanding tumor-promoting signaling. Abnormal glycosylation patterns, often seen in PCs, can drive key processes like immune evasion, metastasis, and cell signaling. Translational research platforms, such as lectin-based assays and glycoproteomics, enable the study of glycan structures on proteins that influence these processes. For instance, the purinergic receptor P2Y2, activated by ATP and linked to invasive behavior in PDAC, may be regulated by specific glycosylation patterns. By targeting the glycosylation machinery that modifies P2Y2 or similar receptors, researchers could inhibit pathways associated with metastasis and create more personalized, effective therapies.
